# In vitro secretion profiles of interleukin (IL)-1beta, IL-6, IL-8, IL-10, and TNF alpha after selective infection with *Escherichia coli* in human fetal membranes

**DOI:** 10.1186/1477-7827-5-46

**Published:** 2007-12-13

**Authors:** Veronica Zaga-Clavellina, Guadalupe Garcia-Lopez, Hector Flores-Herrera, Aurora Espejel-Nuñez, Arturo Flores-Pliego, Diana Soriano-Becerril, Rolando Maida-Claros, Horacio Merchant-Larios, Felipe Vadillo-Ortega

**Affiliations:** 1 Biomedical Research Branch, Instituto Nacional de Perinatologia "Isidro Espinosa de los Reyes", México City, México; 2 Direction of Research, Instituto Nacional de Perinatologia "Isidro Espinosa de los Reyes", México City, México; 3 Microbiology and Parasitology, Instituto Nacional de Perinatologia "Isidro Espinosa de los Reyes", México City, México; 4 Neonatology Branch, Instituto Nacional de Perinatologia "Isidro Espinosa de los Reyes", México City, México; 5 Biomedical Research Institute, Universidad Nacional Autónoma de México, Mexico City, Mexico

## Abstract

**Background:**

Chorioamniotic membranes infection is a pathologic condition in which an abnormal secretion of proinflammatory cytokines halts fetal immune tolerance. The aim of the present study was to evaluate the functional response of human chorioamniotic membranes, as well as the individual contribution of the amnion and choriodecidua after stimulation with Escherichia coli, a pathogen associated with preterm labor.

**Methods:**

Explants of chorioamniotic membranes from 10 women (37–40 weeks of gestation) were mounted and cultured in a Transwell system, which allowed us to test the amnion and choriodecidua compartments independently. Escherichia coli (1 × 10 6 CFU/mL) was added to either the amniotic or the choriodecidual regions or both; after a 24-h incubation, the secretion of IL-1beta, IL-6, TNFalpha, IL-8, and IL-10 in both compartments was measured using a specific ELISA. Data were analyzed by Kruskal-Wallis one-way analysis of variance.

**Results:**

After stimulation with Escherichia coli, the choriodecidua compartment showed an increase in the secretion of IL-1beta (21-fold), IL-6 (2-fold), IL-8 (6-fold), and IL-10 (37-fold), regardless of which side of the membrane was stimulated; TNFalpha secretion augmented (22-fold) also but only when the stimulus was applied simultaneously to both sides. When the amnion was stimulated directly, the level of IL-1beta (13-fold) rose significantly; however, the increase in IL-8 secretion was larger (20-fold), regardless of the primary site of infection. TNFalpha secretion in the amnion compartment rose markedly only when Escherichia coli was simultaneously applied to both sides.

**Conclusion:**

Selective stimulation of fetal membranes with Escherichia coli results in a differential production of IL-1beta, IL-6, TNFalpha, IL-8, and IL-10. These tissues were less responsive when the amnion side was stimulated. This is in agreement with the hypothesis that the choriodecidua may play a primary role during an ascending intrauterine infection, being the main barrier to progression of the infection into the amniotic cavity. Therefore, the tissue-specific capacities for the secretion of these immune modulators could be a determining factor for the degree of severity of the inflammation process in fetal membranes.

## Background

Pregnancy is the result of a fine immunological privilege that allows the fetus to co-habit the maternal uterus, preventing rejection of the fetal allograft [1]. There are epidemiologic and experimental data indicating that intrauterine infection is a pathological condition that disrupts this privilege [2,3] and is the main etiological factor for the development of Premature Rupture of Membranes (PROM). This pathology complicates about 30–40% of all preterm births [4] and 10% of all pregnancies [5].

Human chorioamniotic membranes form a complex multi-laminated tissue constituted by the amnion whose epithelium is in contact with the amniotic fluid and the choriodecidua that is formed by trophoblasts inter-digitized with the maternal decidua [5,6]. During an ascending infection, originated in the cervico-vaginal region [7-9], the choriodecidua is the first-line barrier in contact with the pathogens that can cross the membranes and infect the amnion and the amniotic fluid [10]. In response, there is an abnormal production of proinflammatory cytokines, such as interleukin (IL)-1β, tumor necrosis factor alpha (TNFα), IL-6, and IL-8 in the extra-embryonic tissues, i.e., placenta [9] and chorioamniotic membranes [11-14].

In a previous work, using an *ex-vivo *model, we demonstrated that *in vitro *stimulation with lipopolysaccharide (LPS) and *Streptococcus agalactiae *induced a differential response in IL-1β and TNFα secretion by the amnion and choriodecidual tissues [13]. Using the same model, the present study was aimed at: 1) Investigating the secretory profiles of IL-1β, IL-6, TNFα, IL-8, and IL-10 by human chorioamniotic membranes after stimulation with *Escherichia coli*, a common pathogen in cervical-vaginal infection in humans associated with pregnancy losses [8,15], fetal cardiac dysfunction [16], neurological injury in preterm infants [17], as well as with preterm delivery and PROM [18]. 2) Disclosing whether or not a specific differential contribution by each membrane exists.

## Methods

The 10 pregnant women (37–40 weeks of gestation) studied were from an urban area of Mexico City, 22–35 years old, previously normotensive, without history of diabetes mellitus, thyroid, liver or chronic renal disease, cared for at the Obstetrics Outpatient Service of the Instituto Nacional de Perinatologia. All women had uneventful pregnancies, without evidence of active labor and with neither clinical nor microbiological signs of chorioamnionitis or of lower genital tract infection. Samples were obtained after delivery by elective cesarean section.

All women provided written, informed consent, before collection of samples. The institutional Review Board approved the protocol and the collection and use of the samples and the study was conducted according to the *Guidelines on the Practice of Ethical Committees in Medical Research *(3^rd ^ed) issued by the Royal College of Physicians of London.

### Fetal membrane explants culture

**Figure 1:**
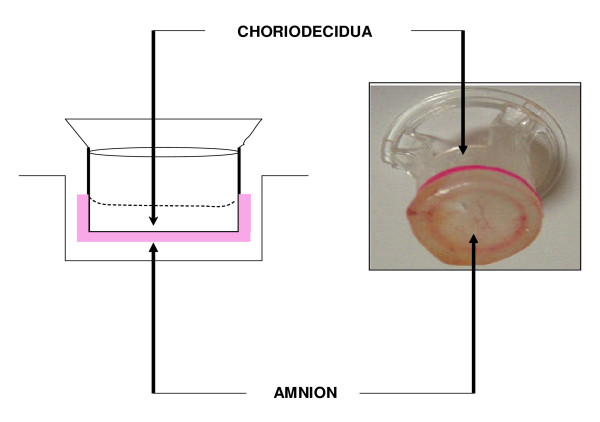
**Experimental model**. The fetal membranes are held together with silicone rubber rings in the upper chamber of a Transwell^® ^device. In this system, the choriodecidua region delimits the upper chamber and the amnion region, the lower chamber.

The chorioamniotic membranes were cut at a distance of 5–6 cm from the placental disc, transported to the laboratory in sterile Dulbecco Modified Eagle Medium (DMEM; Gibco BRL, Bethesda, MD), and rinsed in sterile saline solution to remove adherent blood clots. Segments representing all zones of membranes were manually cut into 18 mm diameter discs and held together with silicone rubber rings to be placed on the upper chamber of a Transwell^® ^system (CORNING, New York, NY) from which the original polycarbonate membrane had been previously removed. In this model the upper chamber of the Transwell^® ^system is delimited by choriodecidual tissue and the lower chamber by amniotic tissue, which allowed testing the two compartments independently (Figure 1). A detailed description and validation of this model has been published previously [13]. One milliliter of DMEM (Gibco BRL), supplemented with 10% fetal calf serum (FCS), 1 mM sodium pyruvate, and 1× antibiotic-antimycotic solution (penicillin 100 U/mL, streptomycin 100 μg/mL) (DMEM-FCS) was added to each compartment. The mounted explants were placed in a 12-well tissue culture plate (CORNING, New York, NY) and incubated in 5% CO_2 _at 37°C for 24 h.

### Explants stimulation

To stabilize the explants after manipulation, they were pre-incubated for 24 h in the medium (DMEM-FCS). Subsequently, the explants were washed with saline solution to remove FCS and the remainder of the medium was changed to DMEM with 0.2% lactalbumin hydrolysate (Gibco BRL) and co-incubated with 1 × 10^6 ^colony forming units (CFU) of *Escherichia coli *isolated from cervicovaginal exudates.

Four Each experiment (n = 10) included the following set of chambers, in triplicate: Basal (n = 10), control membranes in which only the medium culture was added to the compartments; Choriodecidua (n = 10),*Escherichia coli *was added only to the choriodecidua side; Amnion(n = 10),*E. coli *was added only to the amniotic compartment; Both(n = 10), the bacterium was added simultaneously to both compartments. After 24 h of co-incubation, the medium from the amnion and choriodecidua chambers was collected and centrifuged at 5,000 rpm, 3 min at 4°C, to precipitate *Escherichia coli*, and the bacterium-free medium of each sample was stored at -70°C until assayed. Protein concentration in all samples was measured according to the Bradford method [19].

### Cytokine assays

IL-1β, TNF-α, IL-6, and IL-10 concentrations were quantified using specific DuoSet^® ^enzyme-linked immunosorbent sandwich assays (ELISA) (R & D Systems, Minneapolis, USA). For the IL-1β assay, a standard curve was developed from 4 to 260 pg/mL and the sensitivity was 2 pg/mL; the TNF-α assay was linear from 15 to 960 pg/mL and sensitivity was 5.0 pg/mL; for the IL-6 assay, the curve was linear from 250 to 8000 pg/mL with a sensitivity of 200 pg/mL; and for the IL-10 assay, a standard curve was developed from 1.25 to 2000 pg/mL, with a sensitivity of 1.0 pg/mL. IL-8 was measured using a commercial kit (Amersham Biosciences, Buckinghamshire, UK) according to manufacturer's instructions. The standard curve was linear from 25.6 to 1000 pg/mL and sensitivity was 5 pg/mL.

The final concentration of each cytokine was expressed per microgram of the total protein concentration of each sample. Intra- and inter-assay coefficients of variation were less than 5%. A rigorous quality control program, including external and internal standards for all cytokines, is followed in our laboratory.

### Statistical analysis

Since the results did not have a normal distribution, comparisons between the experimental groups and the control were performed using the Kruskal-Wallis one-way analysis of variance on rank tests. A *P *< 0.05 was considered significant. The data expressed in the text and figures represent the median and the 95% confidence interval limits (95% CI).

## Results

**Figure 2:**
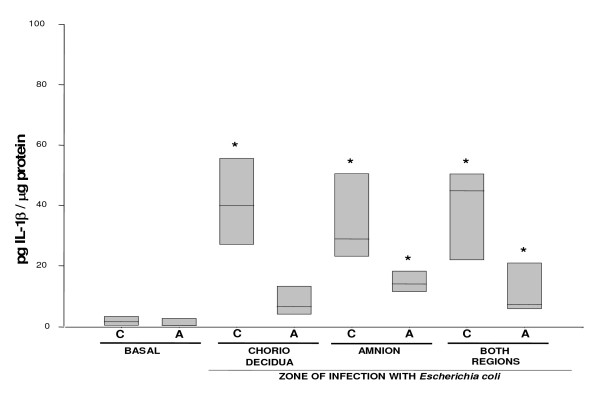
**In vitro secretion of IL-1β in amnion and choriodecidua regions after selective stimulation with *Escherichia coli***. IL-1β secreted to the culture medium after 24 h of infection with 1 × 10^6 ^CFU of *E. coli*. Data were normalized in function of protein concentration (pg/μg protein) and each bar represents the 95% confidence intervals and the median (solid line) of 10 different experiments. Significant difference between basal and stimulated values is indicated (*P < 0.05) C. Choriodecidua; A. Amnion.

Basal secretion of IL-1β was similar in both the amniotic and the choriodecidua compartments (0.8 [0.30–2.89] and 1.73 [0.12–3.27] pg/μg protein, respectively); it increased markedly in the choriodecidua tissue (P < 0.05) regardless of whether *Escherichia coli *was applied directly to either the choriodecidua compartment (39.95 [26.9–55.6] pg/μg protein) or the amniotic compartment (28.9 [23.0–50.5] pg/μg protein), or simultaneously to both compartments (44.87 [21.87–50.5] pg/μg protein). However, IL-1β secreted by the amnion rose slightly only when the bacterium was directly applied to this compartment (13.9 [11.2–18.3] pg/μg protein) (Figure 2).

**Figure 3:**
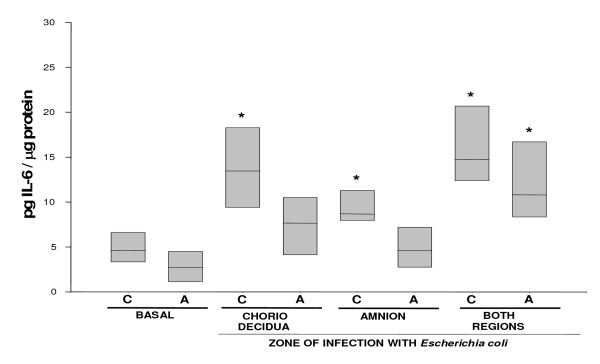
**In vitro secretion of IL-6 in amnion and choriodecidua regions after selective infection with *Escherichia coli***. IL-6 secreted to the culture medium after 24 h of infection with 1 × 10^6^CFU of *E. coli*. Data were normalized in function of protein concentration (pg/μg protein) and each bar represents the 95% confidence intervals and the median (solid line) of 10 different experiments. Significant difference between basal and stimulated values is indicated (*P < 0.05) C. Choriodecidua; A. Amnion.

**Figure 4:**
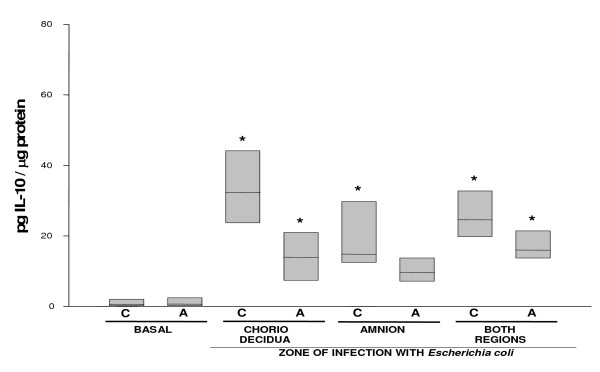
**In vitro secretion of IL-10 in amnion and choriodecidua regions after selective infection with *Escherichia coli***. IL-10 secreted to the culture medium after 24 h of infection with 1 × 10^6^CFU of *E. coli*. Data were normalized in function of protein concentration (pg/μg protein) and each bar represents the 95% confidence intervals and the median (solid line) of 10 different experiments. Significant difference between basal and stimulated values is indicated (*P < 0.05) C. Choriodecidua; A. Amnion.

IL-6 (Figure 3) and IL-10 (Figure 4) showed a similar secretion profile, with the choriodecidua as the most active tissue after *Escherichia coli *stimulation whether applied directly or indirectly to this membrane (P < 0.05). In both cases, the largest response was observed when the stimulus was applied directly in the choriodecidual zone (13.45 [9.39–18.3] and 32.38 [23.6–44.2] pg/μg protein, respectively). Again, the amnion showed only a mild but significant increase in the secretion of IL-6 and IL-10 (10.8 [8.3–16.75] and 15.97 [13.02–21.49] pg/μg protein, respectively).

**Figure 5:**
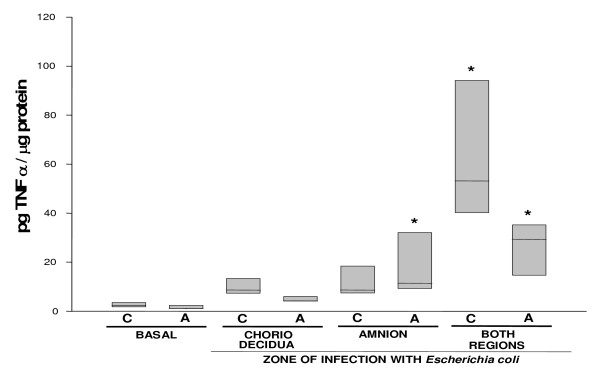
**In vitro secretion of TNF-α in amnion and choriodecidua regions after selective infection with *Escherichia coli***. TNF-α secreted to the culture medium after 24 h of infection with 1 × 10^6 ^CFU of *E. coli*. Data were normalized in function of protein concentration (pg/μg protein) and each bar represents the 95% confidence intervals and the median (solid line) of 10 different experiments. Significant difference between basal and stimulated values is indicated (*P < 0.05) C. Choriodecidua; A. Amnion.

The basal secretion of TNFα by the amnion and choriodecidua was similar (Figure 5). However, the secretion by the choriodecidua tissue showed a 22-fold increase (53.15 [40.0–94.2] pg/μg protein) only when the *Escherichia coli *stimulus was applied simultaneously to both membranes (P < 0.05), while the amnion showed a 25-fold increase (P < 0.05) (29.2 [14.5–35.3] pg/μg protein) (Figure 5).

**Figure 6:**
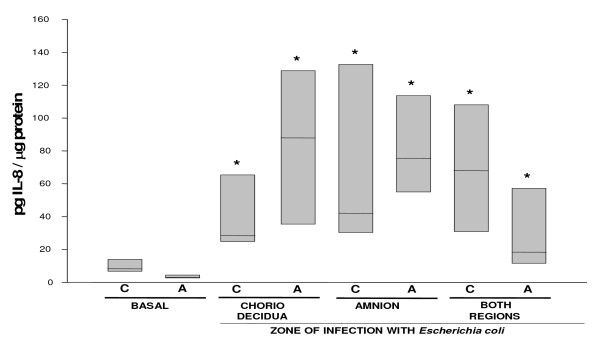
**In vitro secretion of IL-8 in amnion and choriodecidua regions after selective infection with *Escherichia coli***. IL-8 secreted to the culture medium after 24 h of infection with 1 × 10^6^CFU of *E. coli*. Data were normalized in function of protein concentration (pg/μg protein) and each bar represents the 95% confidence intervals and the median (solid line) of 10 different experiments. Significant difference between basal and stimulated values is indicated (*P < 0.05) C. Choriodecidua; A. Amnion.

The secretion profile of IL-8 was different from that of the other cytokines. Regardless of the primary side of stimulation with *Escherichia coli*, the secretion was markedly increased in both compartments; however, the amnion was the most active region with a 19-fold increase (P < 0.05) in comparison with its basal concentration (Figure 6).

## Discussion

In the present study we used a previously published *ex-vivo *model that resembles the presence of two independent compartments with a fully functional chorioamnion; its upper chamber is delimited by the choriodecidua and the lower chamber by the amnion, allowing us to test both compartments simultaneously [13]. We included three different conditions of membrane stimulation with *Escherichia coli*, resembling clinical scenarios. 1) Microorganisms arrive to the choriodecidua through ascendant colonization (choriodecidua stimulation), 2) bacteria reach the amnion side by iatrogenic introduction during amniocentesis (amnion stimulation), and 3) bacteria are in contact with both sides of the membranes at late stages of colonization, which enables them to cross the membranes.

With this system, we were able to show that the basal secretions of IL-1β, IL-6, IL-10, and IL-8 by the choriodecidual tissue increased significantly (P < 0.05) in response to *Escherichia coli *stimulation, regardless of whether the stimulus was applied directly to this region, the amnion, or to both regions simultaneously. In addition, TNFα increased markedly in both the choriodecidua and the amnion sides when the bacterium was applied to both membranes at the same time. IL-1β rose mildly when the amnion was directly stimulated by *Escherichia coli *whereas IL-8 secretion rose markedly in the amnion regardless of the primary side stimulated. A marked rise in TNFα secretion in the amnion side was also observed but only when the bacterium was simultaneously applied to the amnion and choriodecidua tissues.

Previous works have evidenced that inoculation of the amniotic cavity with *Escherichia coli *induces a toxic response, characterized by the overproduction of pro-inflammatory cytokines [20,21], such as IL-1β, a potent immunomodulator able to induce preterm labor after its experimental infusion in pregnant rhesus monkeys [22]. However, the used animal models do not allow characterizing the particular and specific contribution of each of the chorioamniotic membranes in this response and cannot be compared with our results. In addition, we demonstrated that *in vitro *stimulation of chorioamniotic membranes with lipopolysaccharide (LPS) and *Streptococcus agalactiae *induces IL-1β increase in the choriodecidua region [13].

On the other hand, the IL-6 concentration in the amniotic fluid is considered a marker of intra-amniotic inflammation and is frequently associated with an infectious process in either the amniotic fluid or the chorioamniotic space [23,24]. Previous evidence has demonstrated that the concentration of this cytokine is increased in human decidua [25] and chorion cells [26] after treatment with IL-1β and TNFα.

Similar findings have been obtained with IL-10, an anti-inflammatory cytokine, to which a role as therapeutic factor in preterm labor has been ascribed [27] and whose concentration increases in patients with preterm labor associated with intrauterine infection [28].

The differential secretion capacities of pro-inflammatory cytokines by the amnion and the choriodecidua after *Escherichia coli *stimulation here reported suggest the existence of a complex interactive regulation network. There is evidence indicating that IL-1β [29], TNFα [30], IL-6 [31], and IL-10 [32], in different gestational tissues, are able to induce biosynthesis and secretion of prostaglandin E_2 _(PGE_2_) and PGF_2α_; these uterotonic factors play key roles in the onset and progression of labor [33] in normal and pathological conditions. In a previous work, we demonstrated that the amnion's epithelium is the main source of PGE_2 _secretion after stimulation with *Candida albicans*, yeast that has been associated with cervico-vaginal infections [14].

Under the experimental conditions used in our study, it was clear that IL-1β, IL-6, and IL-10 were mainly secreted to the choriodecidual compartment regardless of the primary zone stimulated with *Escherichia coli*. Interestingly, when the amnion was primarily stimulated, its contribution to increase the secretion of theses cytokines was small if any; whereas the secretion of theses cytokines by the choriodecidual region was significantly increased. This observation suggests the existence of a communication or "cross-talk" between both regions and, thus, it is possible that the amnion's epithelium might be an important indirect factor in the whole cytokine response.

On the other hand, TNFα, whose negative effects on the course of pregnancy have been characterized [34,35], increases in the amniotic fluid of women with preterm labor and intra-amniotic infection [30]. There is also experimental evidence demonstrating that the administration of a TNFα bolus to pregnant animals causes profuse hemorrhage and pregnancy termination [36,37]. In our model, the increase in TNFα secretion in both the amnion and choriodecidua compartments was significant (P < 0.05) only after simultaneous stimulation of both membrane sides. A possible interpretation of the biological significance of these findings is that, since TNFα is a pro-inflammatory cytokine with major immuno-toxic properties, the chorioamniotic membrane coordinates its secretion in response to a very complicated infectious scenario, as represented by chorioamnionitis, in which both the fetal and maternal sides are insulted by an infectious agent.

It is possible that the secretion of IL-1β, IL-6, IL-10, and TNFα in the choriodecidua region after infection with *Escherichia coli *would favor their trans-membranal translocation to the amnion [38] and thereby exert their effect on the whole membrane.

The IL-8 secretion pattern was also interesting, because both the amnion and the choriodecidua were active in the secretion of this chemokine. This ubiquitous production might play a key role in the recruitment and activation of professional cells of the immune system, such as neutrophils whose migration toward the cervix, the placenta, and chorioamniotic membranes is a clinical/histological characteristic of infection/inflammation [39,40]. The present results show that the IL-8 response in the amnion was mild as compared to the choriodecidua region, which is the first tissue to be colonized by the microbial pathogen during an ascending intrauterine infection and is the main barrier to progression of infection into the amniotic cavity. Therefore, the tissue-specific capacities could be important factors in determining the severity of the inflammation in fetal membranes infected with *Escherichia coli*.

It is tempting to hypothesize that *Escherichia coli *infection of the chorioamniotic membrane may induce a precocious onset of the overproduction of pro-inflammatory cytokines (an "anticipation" of the normal parturition cascade?), leading to PROM and preterm labor.

## Conclusion

Our results demonstrated that fetal membranes respond differentially to *Escherichia coli *infection. The amnion and choriodecidual cellular populations display a cooperative and bidirectional communication to secret different immunologic modulators, such as Il-1β, TNFα, IL-6, IL-8, and IL-10.

The choriodecidua is the most responsive region to the infection, as it is the first tissue to be colonized by the microbial pathogen during an ascending intrauterine infection and it is the main barrier to progression of the infection into the amniotic cavity. Therefore, the tissue-specific capacities of this region to secrete different proinflammatory cytokines are crucial factors for determining the severity of the inflammation process of fetal membranes.

## Authors' contributions

AEN, AFP, DSB and RMC carried out samples collection, ELISA assays and microbiologic control. GGL carried out culture membranes and stimulation with bacterium. HFH coordinated data collection and provided statistical analysis. VZC, FVO and HML participate in the design of the study, data analysis and manuscript preparation. All authors read and approved the final manuscript.

## References

[B1] Thellin O, Coumans B, Zorzi W, Igout A, Heinen E (2000). Tolerance to the foeto-placental "graft": ten ways to support a child for nine months. Curr Opin Immunol.

[B2] Peltier MR (2003). Immunology of term and preterm labor. Reprod Biol Endocrinol.

[B3] Makhseed M, Raghupathy R, El-Shazly S, Azizieh F, Al-Harmi JA, Al-Azemi MMK (2003). Pro-inflammmatory maternal cytokines profile in preterm delivery. Am J Reprod Immunol.

[B4] Newton ER (2005). Preterm labor, preterm premature rupture of membranes and chorioamnionitis. Clin Perinatol.

[B5] Parry S, Strauss JF (1998). Premature rupture of the fetal membranes. N Engl J Med.

[B6] Malak TM, Ockleford CD, Bell SC, Dalgleish R, Bright N, Macvicar J (1993). Confocal immunofluorescence localization of collagen types I, III, IV, V and VI and their ultraestructural organization in term human fetal membranes. Placenta.

[B7] Romero R, Espinoza J, Goncalves LF, Kusanovic JP, Fiel LA, Nien JK (2006). Inflammation in preterm and term labour and delivery. Sem Fetal Neonatal Med.

[B8] Moyo SR, Tswana SA, Nystrom L, Bergstrom S, Blomerberg J, Ljungh A (1995). Intrauterine death and infections during pregnancy. Int J Gynaecol Obstet.

[B9] El-Shazly S, Machseed M, Azizieh F, Raghupathy R (2004). Increased expression of pro-inflammatory cytokines in placentas of women undergoing spontaneous preterm delivery or premature rupture of membranes. Am J Reprod Immunol.

[B10] Romero R, Mazor M (1988). Infection and preterm labor. Clin Obstet Gynecol.

[B11] Schoonmaker JN, Lawellin DW, Lunt B, McGregor JA (1989). Bacteria and inflammatory cells reduce chorioamniotic membrane integrity and tensile strength. Obstet Gynecol.

[B12] Tashima LS, Millar LK, Bryant-Greenwood GD (1999). Genes upregulated in human fetal membranes by infection or labor. Obstet Gynecol.

[B13] Zaga V, Estrada-Gutierrez G, Beltran-Montoya J, Maida-Claros R, Lopez-Vancell R, Vadillo-Ortega F (2004). Secretion of interleukin-1 beta and tumor necrosis factor alpha by whole fetal membranes depends on initial interactions of amnion or chorion with lipopolysaccharides or group B streptococci. Biol Reprod.

[B14] Zaga-Clavellina V, García-López G, Estrada-Gutierrrez G, Martínez-Flores A, Maida-Claros R, Beltrán-Montoya J, Vadillo-Ortega F (2005). Incubation of human chorioamniotic membranes with Candida albicans induces differential synthesis and secretion of interleukin-1β, inteleukin-6, prostaglandin E2, and 92 kDa type IV collagenase. Mycoses.

[B15] Deb k, Chaturvedi MM, Jaiswal YK (2004). Comprehending the role of LPS in Gram-negative bacterial vaginosis: ogling into the causes of unfulfilled child-wish. Arch Gynecol Obstet.

[B16] Rounioja S, Rasanen J, Glumoff V, Ojaniemi M, Makikallio K, Hallman M (2003). Intra-amniotic lipopolysaccharide leads to fetal cardiac dysfunction. A mouse model for fetal inflammatory response. Cardiovasc Res.

[B17] Dunkan JR, Cock ML, Scheerlinck JP, Westcott KT, McLean C, Harding R, Rees SM (2002). White matter injury after repeated endotoxin exposure in the preterm ovine fetus. Pediatr Res.

[B18] Romero R, Mazor M (1988). Infection and preterm labor. Clin Obstet Gynecol.

[B19] Stoscheck CM (1990). Quantitation of Protein. Methods in Enzymology.

[B20] Hirsch E, Saotomo I, Hirsh D (1995). A model of intraturerine infection and preterm delivery in mice. Am J Obstet Gynecol.

[B21] Reznikov LL, Fantuzzi G, Selzman CH, Shames BD, Barton HA, Bell H, McGregor JA, Dinarello CA (1999). Utilization of endoscopic inoculation in a mouse model of intrauterine infection-induced preterm birth; role of interleukin 1β. Biol Reprod.

[B22] Vadillo-Ortega F, Sadowsky DW, Haluska GJ, Hernández-Guerrero C, Guevara-Silva Rebeca, Gravett MG, Novy MJ (2002). Identification of matrix metalloproteinase-9 in amniotic fluid and amniochorion in spontaneous labor and after experimental intrauterine infection or interleukin-1β infusion in pregnant rhesus monkeys. Am J Obstet Gynecol.

[B23] Romero R, Avila C, Santhanam U, Sehgal PB (1990). Amniotic fluid interleukin-6 in preterm labor: Association with labor. J Clin Invest.

[B24] Hsu CD, Meaddough E, Aversa K, Hong SF, Lu LC, Jones DC, Copel JA (1998). Elevated amniotic fluid levels of leukemia inhibitory factor, interleukin 6, and interleukin 8 in intra-amniotic infection. Am J Obstet Gynecol.

[B25] Dudley DJ, Trautman MS, Araneo BA, Edwin SS, Mitchell MD (1992). Decidual cell biosynthesis of interleukin-6: regulation by inflammatory cytokines. J Clin Endocrinol Metab.

[B26] Dudley DJ, Trautman MS, Edwin SS, Ludin-Schiller S, Mitchel MD (1992). Biosynthesis of interleukin-6 by cultured human chorion laeve cells: regulation by cytokines. J Clin Endocrinol Metab.

[B27] Sato TA, Keelan JA, Mitchell MD (2003). Critical paracrine interactions between TNFα and IL-10 regulate lipopolysaccharide-stimulated human choriodecidual cytokine and prostaglandin E_2 _production. J Immnunol.

[B28] Grieg PC, Herbert WNP, Robinnette BL, Teot LA (1995). Amniotic fluid interleukin-10 concentrations increase through pregnancy and are elevated in patients with preterm labor associated with intrauterine infection. Am J Obstet Gynecol.

[B29] Romero R, Brody DT, Oyarzun E, Mazor M, Wu YK, Hobbings JC, Durum SK (1989). Infection and labor. III. Interleukin-1: A signal for the onset of labor. Am J Obstet Gynecol.

[B30] Romero R, Manogue KR, Mitchell MD, Wu YK, Oyarzun E, Hobbins JC, Cerami A (1989). Infection and preterm labor. IV. Cachectin-tumor necrosis factor in the amniotic fluid of women with intraamniotic infection and preterm labor. Am J Obstet Gynecol.

[B31] Mitchel MD, Dudley DJ, Edwin SS, Schiller SL (1991). Interleukin-6 stimulates prostaglandin production by human amnion and decidual cells. Eur J Pharmacol.

[B32] Mitchell MD, Simpson KL, Keelan JA (2004). Paradoxical proinflammatory actions of interleukin-10 in human amnion: potential role in term and preterm labor. J Clin Endocrinol Metab.

[B33] Okazaki T, Casey ML, Okita J, MacDonald PC, Johnson JM (1981). Initiation of parturition XII. Biosynthesis and metabolism of prostaglandin in human fetal membranes and uterine decidua. Am J Obstet Gynecol.

[B34] Romero R, Mazor M, Sepulveda W, Avila C, Copeland D, Williams J (1992). Tumor necrosis factor in preterm and term labor. Am J Obstet Gynecol.

[B35] Hunt JS, Chen HL, Miller L (1996). Tumor necrosis factors: pivotal components of pregnancy?. Biol Reprod.

[B36] Zahl PA, Bjerknes C (1943). Induction of decidua-placental hemorrhage in mice by the endotoxins in certain gram-negative bacteria. Proc Soc Exp Biol Med.

[B37] Silver RM, Lohner WS, Daynes RA, Mitchell MD, Branch DW (1994). Lipopolysaccharide – induced fetal death: the role of tumor-necrosis factor alpha. Biol reprod.

[B38] Kent ASH, Sullivan MHF, Elder MG (1994). Transfer of cytokines through human fetal membranes. J Reprod Fertil.

[B39] Steinborn A, Kühnert M, Halberstadt E (1996). Immunmodulating cytokines induce term and preterm parturition. J Perinat Med.

[B40] Keelan JA, Marvin KW, Sato TA, Coleman M, McCowan LME, Mitchell MD (1999). Cytokine abundance in placental tissues: Evidence of inflammatory activation in gestation membranes with term and preterm parturition. Am J Obstet Gynecol.

